# Genome-Wide Identification of *WOX* Gene Family in *Chimonanthus praecox* and a Functional Analysis of *CpWUS*

**DOI:** 10.3390/plants14071144

**Published:** 2025-04-07

**Authors:** Huafeng Wu, Bin Liu, Yinzhu Cao, Guanpeng Ma, Xiaowen Zheng, Haoxiang Zhu, Shunzhao Sui

**Affiliations:** 1 Chongqing Engineering Research Center for Floriculture, Key Laboratory of Agricultural Biosafety and Green Production of Upper Yangtze River (Ministry of Education), College of Horticulture and Landscape Architecture, Southwest University, Chongqing 400715, China; woo925784127@163.com (H.W.); 15705983137@163.com (B.L.); yinzhu202108@163.com (Y.C.); guanpengma@163.com (G.M.); wennnn_n@163.com (X.Z.); 2 Institute of Horticulture, Guizhou Academy of Agricultural Sciences, Guiyang 550006, China; 3 College of Horticulture and Landscape Architecture, Southwest University, Chongqing 400715, China; zhuhx8910@swu.edu.cn

**Keywords:** *Chimonanthus praecox*, WOX family, *CpWUS*, flowering, root development

## Abstract

*Chimonanthus praecox*, also known as wintersweet, is a traditional ornamental plant in China. It blooms during the cold winter months and emits a long-lasting fragrance. The WUSCHEL-related homeobox (WOX) transcription factor family is a plant-specific family of homeodomain (HD) transcription factors that plays diverse roles in plant development. We identified 13 *WOX* family genes (*CpWOX1–CpWOX12* and *CpWUS*) and systematically analysed their physicochemical properties, evolutionary relationships, conserved domains, and expression regulation characteristics. The subcellular localization prediction indicates that all CpWOX proteins are localized in the nucleus and contain a conserved homeobox domain, with the WUS clade specifically containing a WUS-box motif. Phylogenetic analysis revealed that these genes are divided into three evolutionary branches: the WUS, ancient, and intermediate clades. Promoter analysis suggests that *CpWOX* genes may be involved in hormone responses, abiotic stress, developmental regulation, and encodes a nuclear-localised protein with self-activating activity. It is highly expressed in the stamen and root and is induced by low and high temperatures, salt stress, and methyl jasmonate. This study revealed the evolutionary characteristics of the WOX family genes in wintersweet and the function of *CpWUS* in regulating flowering time and root development, providing a theoretical basis for understanding the developmental regulatory mechanisms in wintersweet.

## 1. Introduction

The WUSCHEL-related homeobox (WOX) transcription factor family is a plant-specific family of homeodomain (HD) transcription factors that play diverse roles in plant development, with crucial regulatory functions [1,2]. The *WOX* gene family was first identified in *Arabidopsis thaliana* and consists of 15 members (WUS, WOX1-WOX14) [3,4]. Subsequently, the WOX family of proteins has been identified in many plants, including poplar [5], rose [6], *Citrus sinensis* [7], and rice [8]. Phylogenetic analysis indicated that WOX proteins are divided into three evolutionary branches: the WUS clade, which is the modern clade; the intermediate clade; and the ancient clade. The WUS clade is unique to higher seed plants, such as rice, sorghum, maize, Arabidopsis, and poplar [8,9]. The typical structural feature of proteins encoded by the WOX gene family is the presence of a homeodomain, which consists of 65 amino acid residues (66 in WUS) and forms a “helix–loop–helix–turn–helix” spatial structure. This structure is highly conserved across species and is crucial for functional integrity during plant growth and development [10,11]. Members of the WUS clade contain a WUS-box motif (T-L-X-L-F-P-X-X, where X can be any amino acid) [4], while the intermediate and ancient clades lack this motif. The WUS-box motif plays an important role in floral organ development and maintains the balance between stem cell division and differentiation [12]. Additionally, some WOX proteins contain an EAR motif at the carboxyl terminus, which functions in transcriptional repression [9,13].

Flowers are important functional organs for reproduction and evolution [14]. The floral organs of plants originate from the floral meristem (FM), the dynamic maintenance of which is crucial for flower development [15,16]. During flower development, WOX transcription factors play an important role in key life activities such as embryo development, floral organ formation, and overall flower development [17,18]. In *Arabidopsis*, AtWUS maintains stem cell activity in the central zone (CZ) of the shoot and floral meristems, ensuring normal differentiation of the shoot apical meristem and floral organs [3]. When *WUS* is mutated, the FM is prematurely depleted, leading to impaired floral organ development, indicating that WUS is essential for maintaining the organogenesis of FM [3,19,20]. Additionally, *AtWUS* is involved in anther and ovarian development. In the early stages in *Arabidopsis*, *WUS* is expressed in the anthers and is essential for anther development [21]. *WUS* regulates the expression of the MADS-box family C-class member AGAMOUS (AG) and interacts with AG to jointly regulate the fate of the floral meristem and determine the identity of the inner whorl floral organs [22,23,24]. CsWUS and CsLFY interact to regulate the maintenance of shoot apical meristem and promote flower development in cucumber by activating *CsAP3* and *CUM1* [25]. *AtWOX13* and *AtWOX14* are expressed in the gynoecium, stamen, and early stages of flower development and regulate the transition to flowering and flower development in *Arabidopsis* [26]. *AtWOX3* (*PRS*) regulates the development of lateral axis of *Arabidopsis* flower and affects its morphology [27], and *WOX1* plays an important role in the development of petals and sepals [28]. In tomatoes, *SlWUS* influences the morphological formation of floral organs [29]; *WOX3* in *Medicago truncatula* [30] plays a crucial role in regulating the floral meristem. In petunias, *PtWOX1* maintains flower development, whereas *PtWOX8* regulates inflorescence development [31]. These findings highlight the significant role of WOX transcription factors in plant flower development.

The WOX family proteins play an important role in root development. Root development depends on the maintenance of homeostasis in root apical meristems (RAM). In *Arabidopsis*, *AtWOX5* is highly expressed in the quiescent centre (QC) of the root cap and performs a function similar to that of WUS in the shoot apical meristem (SAM). It regulates the *PLETHORA* (*PLT*) family genes and the auxin signalling pathway to maintain the activity of root stem cells. The *wox5* mutants exhibited a root stem cell depletion phenotype, indicating their critical role in maintaining RAM homeostasis. Notably, regarding the maintenance of stem cells in RAM, *AtWUS* and *AtWOX5* are functionally interchangeable [32,33]. *OsWOX11* responds to environmental signals such as auxins and cytokinins to promote root regeneration and development [34]. *WOX11* and *WOX12* work together to induce the first step in transformation of parenchymal cells in the primordial xylem or nearby cells into root initiation cells [35]. *OsWOX10* and *OsWOX11* play important roles in root primordium development during auxin-mediated processes [36]. In woody plants, *PoWOX4*, *PoWOX5*, *PoWOX11*, and *PoWOX13b* are preferentially expressed in the roots during the early stages of root primordia formation and may be involved in root development [37]. *RhWOX331* is highly expressed in roots and exogenous indole-3-butyric acid (IBA) promotes the formation of root systems in transgenic *Arabidopsis* [6]. *PtoWOX5a*, *PtoWOX11/12a*, *PtoWUSa*, and *PtoWOX4a* promote adventitious root (AR) regeneration in poplar [38].

*C. praecox* is an ornamental deciduous plant belonging to the genus *Chimonanthus*. As one of the few plants that bloom in winter, *C. praecox* not only provides unique landscape value during the cold season, but also contains abundant aromatic compounds in its flowers, making it widely used in horticulture, essential oil extraction, and the fragrance industry [39]. In recent years, wintersweet has also gained increasing popularity as a woody cut flower. Therefore, studying its flower development is of great significance. Currently, research on the floral development of *C*. *praecox* has made significant progress. *CpFT1* can promote early flowering in *A. thaliana* and may play an important role in breaking dormancy in *C. praecox* [40]. Through the identification of MIKC^C^-type MADS-box family proteins, it is speculated that *CpFUL*, *CpSEPs*, and *CpAGL6s* are involved in dormancy release and bud formation [41]. Overexpression of *CpAGL6* inhibits the expression of *FLC* while promoting the expression of *AP1* and *FT*, thereby accelerating flowering in *Arabidopsis* [42].

Research on various plants has shown that the WOX transcription factor family plays crucial roles in flower and root development. Therefore, investigating the WOX transcription factor family in wintersweet is of great theoretical significance to understand its development. This study used bioinformatics methods to identify *WOX* gene family members in the wintersweet genome and explored their phylogenetic relationships, gene structure, conserved motifs, and chromosomal locations. Additionally, the function of *WUS* in the *WUS* clade was investigated, helping to better understand the role of wintersweet WOX transcription factors in the flowering process and lay the foundation for further analysis of flower development in *C. praecox*.

## 2. Results

### 2.1. Identification and Physicochemical Properties of CpWOX Genes

Using the hidden Markov model (Pf00046) for the homeodomain reported in the Pfam database, WOX proteins were screened using HMMER 3.0 program in wintersweet. The domains of candidate WOX family members were analysed using the NCBI-CD-Search online tool and the hidden Markov model. Only the candidate sequences with complete homeobox domains were retained. A total of 13 *WOX* family genes were identified (Table 1), i.e., Cpra01G00013.1, Cpra02G01741.1, Cpra03G00351.1, Cpra03G01103.1, Cpra04G00358.1, Cpra05G00919.1, Cpra06G01843.1, Cpra08G00857.1, Cpra08G00936.1, Cpra09G00234.1, Cpra09G00715.1, Cpra11G00217.1, and Cpra11G00833.1. These genes were named *CpWOX1–CpWOX12* and *CpWUS* according to their chromosomal positions. Their amino acid lengths ranged from 159 to 360, with molecular weights varying between 18.67 kDa and 37.94 kDa. The theoretical isoelectric points (pI) ranged from 5.25 to 9.05, while the instability indices were between 54.08 and 76.21. The aliphatic indices spanned from 49.11 to 71.7, and the average hydrophilicity values ranged from −0.943 to −0.424. Subcellular localization predictions suggested that all WOX family proteins were localized in the nucleus.

### 2.2. Multiple Sequence Alignment and Phylogenetic Tree Analysis of Wintersweet WOX Protein Family

**Figure 1:**
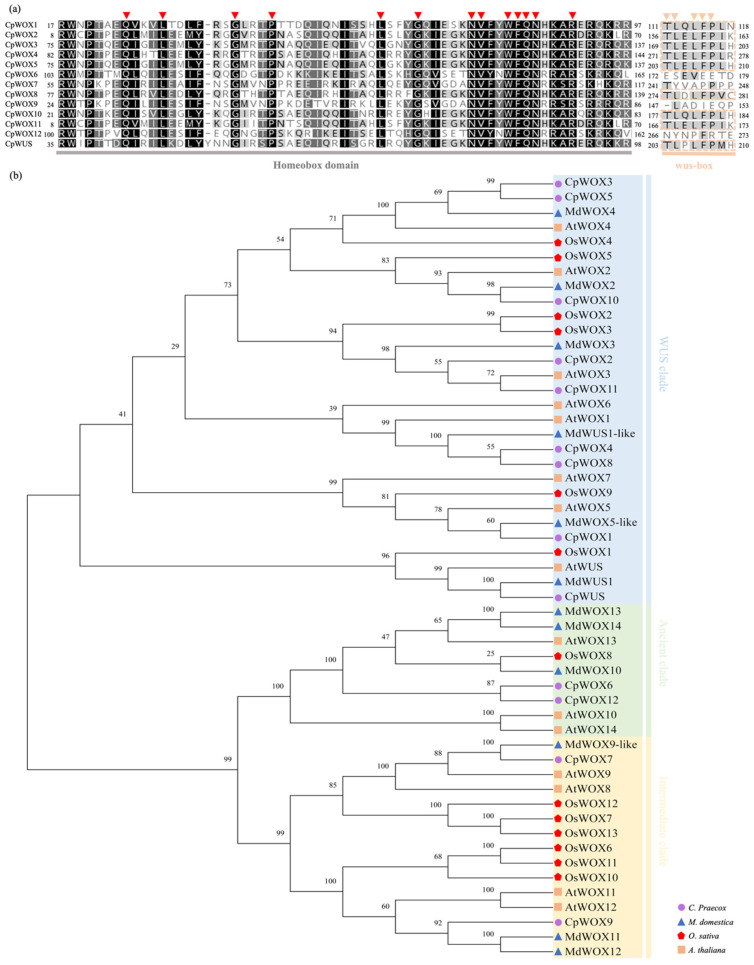
Conserved domain and evolutionary analysis of CpWOX proteins. (**a**) Sequence alignment of the homeobox domain and WUS-box motif of CpWOX proteins, with red arrows indicating the 13 conserved positions of the helix-loop-helix-turn-helix structure, and orange arrows pointing to the conserved positions of the WUS-box motif. (**b**) Phylogenetic tree of WOX family members from wintersweet, apple, rice, and *Arabidopsis*. Phylogenetic tree was constructed using the neighbour-joining (NJ) method, with 1000 bootstrap replicates, using MEGA X.

WOX family members contain a conserved homeodomain structure consisting of 60 amino acids [9]. These 60 amino acids adopt a helix-loop-helix-turn-helix spatial structure, which is highly conserved across various species, playing a crucial role in preserving the functional integrity of the WOX family [43]. The analysis of the wintersweet WOX family protein sequences using DNAMAN 7.0 (Figure 1a) showed that the wintersweet WOX family proteins contained the homeobox domain and included 13 reported conserved sites, including Q and L sites in Helix1, G site in Loop, P and L sites in Helix2, G site in Turn, and N, V, W, F, Q, N, and R sites in helix. Meanwhile, Cpra01G00013.1 (CpWOX1), Cpra02G01741.1 (CpWOX2), Cpra03G00351.1 (CpWOX3), Cpra03G01103.1 (CpWOX4), Cpra04G00358.1 (CpWOX5), Cpra08G00857.1 (CpWOX8), Cpra09G00234.1 (CpWOX10), Cpra09G00715.1 (CpWOX11), and Cpra11G00833.1 (CpWUS) contain the WUS clade-specific WUS-box structure.

A phylogenetic tree was constructed using MEGA X, based on the WOX protein sequences from Arabidopsis [8], rice [8], apple [44], and the identified wintersweet WOX proteins. The results (Figure 1b) showed that the 13 Wintersweet WOX family genes could be divided into three clades. Cpra01G00013.1 (CpWOX1), Cpra02G01741.1 (CpWOX2), Cpra03G00351.1 (CpWOX3), Cpra03G01103.1 (CpWOX4), Cpra04G00358.1 (CpWOX5), Cpra08G00857.1 (CpWOX8), Cpra09G00234.1 (CpWOX10), Cpra09G00715.1 (CpWOX11), and Cpra11G00833.1 (CpWUS) belong to the WUS clade, containing the WUS clade-specific WUS-box motif. Cpra05G00919.1 (CpWOX6) and Cpra11G00217.1 (CpWOX12) belong to the ancient clade, while Cpra06G01843.1 (CpWOX7) and Cpra08G00936.1 (CpWOX9) belong to the intermediate clade.

### 2.3. Conserved Motif, Domain, Gene Structure, and Promoter Cis-Acting Element Analysis of CpWOX

**Figure 2:**
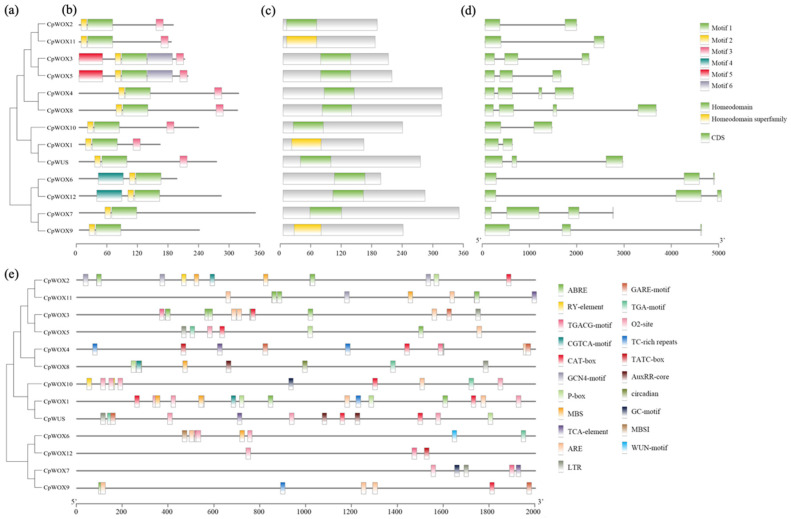
Analysis of conserved motifs, gene structure, and promoter cis-acting elements of CpWOX. (**a**) Phylogenetic tree of CpWOX family. (**b**) Conserved motif. Motifs 1–6 are represented in different coloured boxes, and the box length represents motif length. (**c**) Homeodamain and Homeodamain superfamily conserved domains of CpWOX proteins in wintersweet. (**d**) Intron-exon structure. Green boxes represented exons, and black lines represented introns. (**e**) Cis-acting elements predicted using PlantCARE.

The CpWOX family proteins in wintersweet exhibited six conserved motifs (Figure 2b). CpWOX3 and CpWOX5 contained the most conserved motifs, with six in total, while CpWOX7 and CpWOX9 had only two conserved motifs. Some conserved motifs were unique to specific evolutionary branches, such as motif 3, which is exclusive to the WUS clade and corresponds to the WUS-box sequence, and motif 4, which is present only in the ancient clade. Remarkably, all 13 CpWOX family proteins possess the Homodomain/Homodomain superfamily conserved domain (Figure 2c), and all include motif 1 and motif 2, which are sequences present in the conserved functional domain, homeobox. This suggests that these two motifs are the most highly conserved sequences in the wintersweet WOX family proteins. Gene structure analysis (Figure 2d) shows that the number of exons in the wintersweet WOX family genes ranges from two to four. Promoter cis-acting element prediction results (Figure 2e) revealed 21 types of cis-acting elements in the *CpWOX* family. Hormone-related cis-acting elements mainly include auxins, salicylic acid (SA), methyl jasmonate (MeJA), and gibberellin. Abiotic stress-related elements are mainly involved in responses to low temperatures, drought, and anoxic conditions. Several cis-acting elements are involved in plant defence and stress responses. Notably, some *CpWOX* family members contain cis-acting regulatory elements, such as the CAT-box and GCN4-motif, which are involved in meristem and endosperm expression, and the WUN-motif and RY-element, which are involved in plant wound responses and seed germination regulation.

### 2.4. CpWOX Genes Localisation and Synteny Analysis

**Figure 3:**
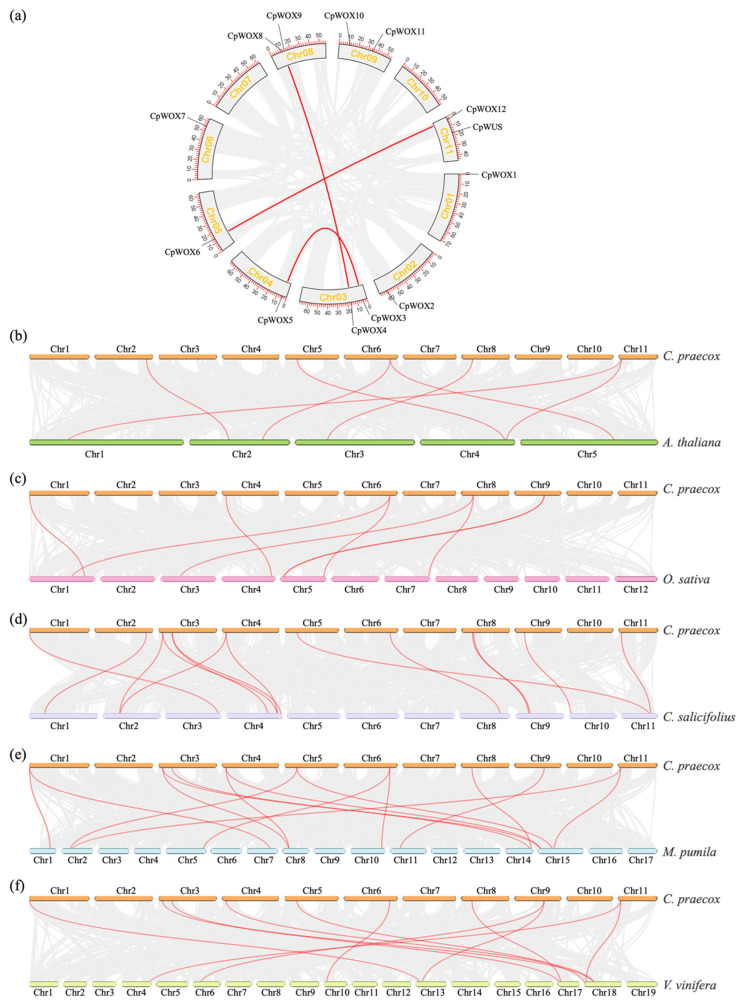
Chromosomal localisation and synteny analysis of *CpWOX* family genes. (**a**) Chromosomal location and synteny analysis of *CpWOX* genes; synteny analysis of wintersweet with Arabidopsis (**b**), rice (**c**), *Chimonanthus salicifolius* (**d**), apple (**e**), and grape (**f**).

Gene localisation results (Figure 3a) showed that *CpWOX* was distributed on all chromosomes, except Chr7 and Chr10. The synteny analysis of the coding genes between wintersweet and *Arabidopsis*, rice, *Chimonanthus salicifolius*, apple, grape, and wintersweet revealed all the gene duplication events. The gene duplication analysis of all coding genes in wintersweet (Figure 3a) revealed that six members of the CpWOX family formed three pairs of syntenic genes. Additionally, CpWOX3/5, CpWOX6/12, and CpWOX4/8 clustered into the same branch, suggesting that the divergence of the paired syntenic genes may have occurred relatively recently. The *CpWOX* family in wintersweet showed synteny with *Arabidopsis* (Figure 3b), rice (Figure 3c), *Chimonanthus salicifolius* (Figure 3d), apple (Figure 3e), and grape (Figure 3f), forming 7, 7, 14, 15, and 11 pairs of syntenic genes, respectively.

### 2.5. CpWUS Sequence Feature Analysis

**Figure 4:**
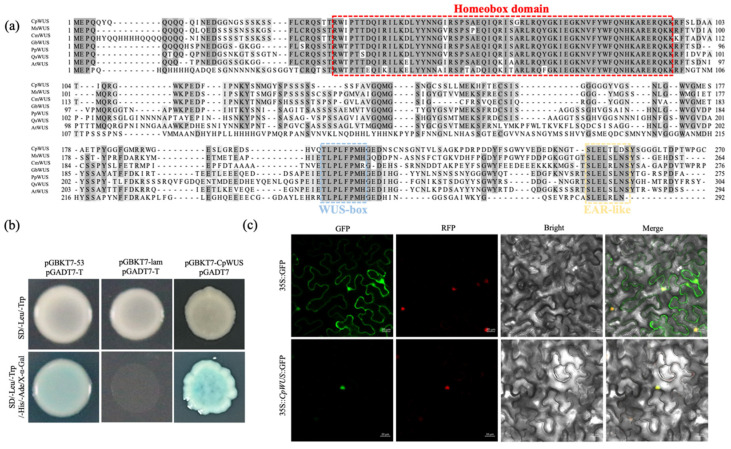
Sequence feature analysis of CpWUS in wintersweet. (**a**) Multiple sequence alignment of CpWUS with *WUS* from *Magnolia sinica*, *Cinnamomum micranthum*, *Gastrolobium bilobum*, *Prunus persica*, *Quillaja saponaria*, and *Arabidopsis*. The Homeobox domain, WUS-box, and EAR-like motif are highlighted in red, blue, and yellow boxes, respectively. (**b**) Transcriptional self-activation activity of CpWUS protein in yeast cells. Co-transformation of pGBKT7-53 and pGADT7-T as positive controls, and pGBKT7-Lam and pGADT7-T as negative controls. (**c**) Subcellular localisation of CpWUS in tobacco epidermal cells.

Sequence analysis showed that the coding sequence (CDS) of CpWUS is 813 bp, encoding 270 amino acid residues. CpWUS contains a homeobox domain, a WUS-box, and an EAR-like motif (Figure 4a). We used a yeast system to analyse the transcriptional activity of CpWUS. The results showed that pGBKT7-WUS and pGADT7 co-transformed into Y2H Gold yeast strain grew normally and turned blue on SD/-Leu/-Trp/-His/-Ade/X-α-Gal medium, indicating that CpWUS has self-activating transcriptional activity (Figure 4b). Further verification of the subcellular localisation of CpWUS (Figure 4c) in tobacco epidermal cells transiently expressing 35S::*CpWUS*::GFP showed that the fluorescent signal was mainly located in the nucleus, indicating that CpWUS predominantly functions in the nucleus.

### 2.6. Expression Characteristics of CpWUS

**Figure 5:**
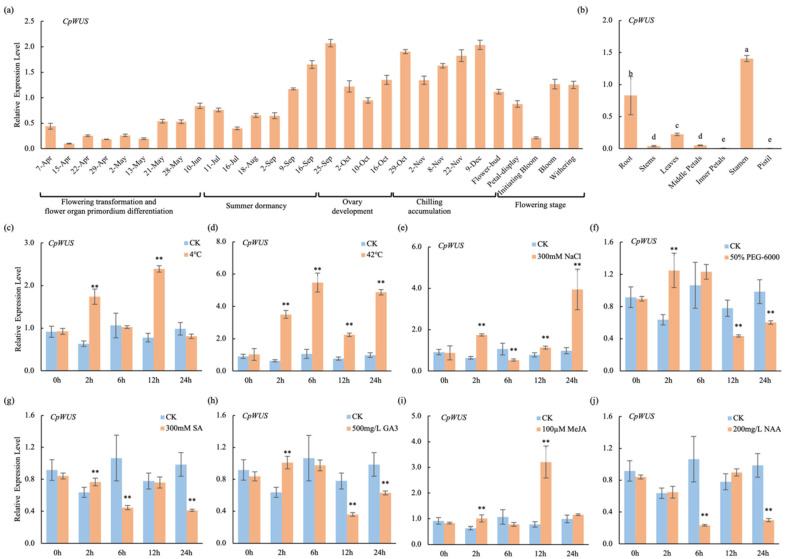
Expression profile analysis of *CpWUS*. (**a**) Expression of *CpWUS* in buds at different flowering stages. (**b**) Tissue-specific expression characteristics of *CpWUS*. Different lowercase letters above bars indicate significant differences. *CpWUS* expression characteristics under treatments of 4 °C (**c**), 42 °C (**d**), 300 mM NaCl (**e**), 50% PEG-6000 (**f**), 300 mM SA (**g**), 500 mg/L GA_3_ (**h**), 100 mM MeJA (**i**), and 200 mg/L NAA (**j**) using a six-leaf stage wintersweet as material. CK refers to untreated wintersweet. ** *p* < 0.01.

During different flowering periods in wintersweet, the expression of *CpWUS* remained relatively low during the transition to flowering and differentiation of floral organ primordia. During summer dormancy, *CpWUS* expression increased. *CpWUS* expression is relatively high during stamen and pistil development and during low-temperature accumulation. *CpWUS* expression decreased during the initial blooming period and gradually increased during the flowering and wilting periods (Figure 5a). qRT-PCR analysis indicated that *CpWUS* expression was higher in the roots. In the wintersweet floral organs, *CpWUS* was highly expressed in the stamens, whereas its expression levels were very low in the pistils and petals (Figure 5b).

To explore the effects of stress and exogenous hormones on *CpWUS* expression, qRT-PCR was used to assess the transcriptional levels of *CpWUS* under different abiotic stresses (4 °C, 42 °C, NaCl, and PEG-6000) and hormone treatments (SA, GA_3_, MeJA, and NAA). The results showed that *CpWUS* expression was induced and peaked at different time points in 4 °C (Figure 5c), 42 °C (Figure 5d), and MeJA (Figure 5i) treatments. For the NaCl (Figure 5e) treatment, the expression level was significantly higher than that of CK at 2, 12, and 24 h, whereas it was significantly lower than that of CK at 6 h. In the PEG-6000 (Figure 5f), SA (Figure 5g), and GA_3_ (Figure 5h) treatments, *CpWUS* was first induced and then inhibited. After NAA treatment (Figure 5j), *CpWUS* expression was suppressed at 6 and 24 h.

### 2.7. CpWUS Regulates Flowering Time in Nicotiana benthamiana

**Figure 6:**
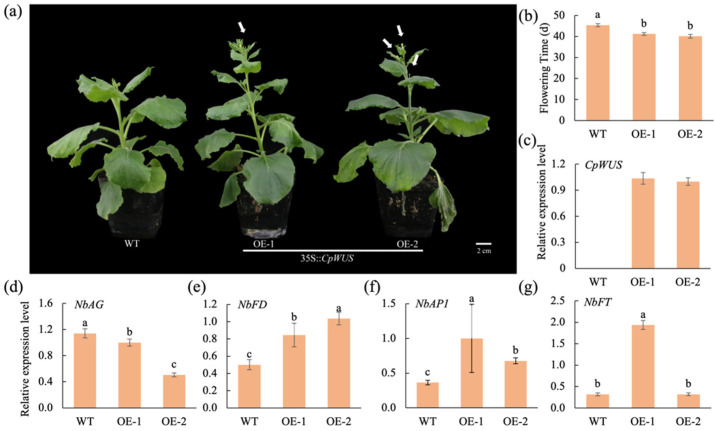
Overexpression of *CpWUS* accelerates flowering in *Nicotiana benthamiana*. (**a**) *CpWUS* transgenic *N. benthamiana* flowers early; (**b**) flowering days of *CpWUS* transgenic *N. benthamiana* and WT; relative expression levels of *CpWUS* (**c**), *NbFD* (**d**), *NbAP1* (**e**), *NbFT* (**f**), and *NbAG* (**g**) in 35S::*CpWUS* transgenic lines and WT. Different lowercase letters indicate significant differences.

To investigate the functional role of *CpWUS*, ectopic overexpression of *CpWUS* under the control of CaMV 35S promoter was performed in *N. benthamiana*. We found that the transgenic *N. benthamiana* lines flowered significantly earlier than the WT plants (Figure 6a–c). Additionally, the expression of flowering-related genes (*NbFT, NbAP1*, and *NbFD*) was significantly upregulated in transgenic *N. benthamiana* plants. Although *WUS* regulates anther development in *Arabidopsis*, no changes have been observed in the number of anthers in *N. benthamiana* plants. However, the overexpression of *CpWUS* inhibited the expression of the MADS-box family C class gene, *AG*, in *N. benthamiana*.

### 2.8. CpWUS Regulates Root Development in Nicotiana benthamian

**Figure 7:**
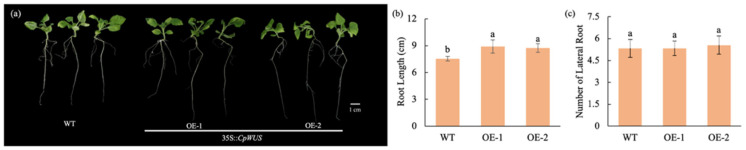
*CpWUS* promotes primary root length in *Nicotiana benthamiana*. (**a**) Root phenotype of *CpWUS* transgenic *N. benthamiana* after 21 days of growth. Root length (**b**) and number of lateral roots (**c**) in *CpWUS* transgenic lines. Different lowercase letters indicate significant differences.

*CpWUS* is highly expressed in the roots, suggesting that it may play an important role in regulating root development. We analysed whether *CpWUS* overexpression affected the root development in *N. benthamiana* (Figure 7). We found that the overexpression of *CpWUS* significantly increased primary root length but did not affect the number of lateral roots.

## 3. Discussion

The WOX transcription factor family is unique to plants, and the proteins they encode are involved in the growth and development of almost all angiosperm organs [13,45]. It mainly consists of three branches: the ancient clade (present in all plants and green algae), the intermediate clade (originating from vascular plants), and the WUS clade (specific to seed plants) [8,9,26]. Many angiosperm WOX genes have been systematically identified and studied using an increasing number of plant genome sequences. In this study, 13 *WOX* gene family members were identified, which were clustered into three branches: the *WUS*, ancient, and intermediate clades. Additionally, we found that specific motifs are required for the functions of subfamilies, such as motif 5, which is specific to the *WUS* clade, and motif 4, which is specific to the ancient clade. Additionally, the WUS clade had the largest number of members, similar to *Arabidopsis* [8], *Oryza sativa* [8], wheat [46], poplar [8], and cotton [47]. The number of *WOX*-type genes varies among species. For example, twenty-three WOX transcription factor-encoding genes have been identified in tobacco K326 [48], whereas only eight have been found in *Pinus massoniana* [49]. The prediction results show that all 13 WOX proteins in *C. praecox* are localized in the nucleus, which is consistent with the typical characteristics of transcription factors. Additionally, their relatively low grand average hydrophobicity value may help maintain the stability of WOX proteins in hydrophilic environments (such as the nucleus) and facilitate their DNA binding and regulatory functions. It is inferred that the difference in the number of WOX family members in different plants is due to gene duplication and adaptation to different environments [50]. Chromosome mapping analysis showed that *CpWOXs* were unevenly distributed across the chromosomes. Except for Chr7 and Chr10, *CpWOXs* were present on all chromosomes. The *CpWOX* gene contains two to four exons. CpWOX contains a highly conserved homodomain consistent with the structural features of WUS genes. Additionally, the WUS clade includes a unique WUS-box domain that aligns with the characteristics of this branch [9]. Studies have shown that WOX genes are involved in the responses of various plants to different abiotic stresses [5,51]. The prediction of cis-acting elements in the promoter (Figure 2d) revealed that *CpWOXs* contain multiple cis-acting elements that respond to stress and hormones, suggesting that they may be involved in the response to abiotic stress. Synteny analysis (Figure 3) revealed that the number of collinear genes between wintersweet and perennial woody plants (such as apple, *Chimonanthus salicifolius*, and grape) was greater than that between annual herbaceous plants (such as *Arabidopsis* and rice), indicating a closer evolutionary relationship between wintersweet and woody plants.

In *Arabidopsis*, *WUS* is primarily expressed in the central region of meristematic tissues, including the SAM, floral meristem, and RAM. Its role is closely associated with maintaining stem cell populations, organ development, and plant growth [19,52]. In the present study, *CpWUS* showed higher expression in the anthers and roots (Figure 5b) and exhibited elevated expression during the late summer dormancy period, during pistil and stamen development, and during the cold accumulation process that breaks dormancy (Figure 5a). This suggests that *CpWUS* plays an important role in wintersweet flower and root development. In Oryza sativa, most *WOX* genes are involved in hormone signalling and abiotic stress responses, such as drought, NaCl, and cold [53]. *SlWOXs* in tomato exhibit strong differential expression patterns under cold, NaCl, and drought stress [54], and overexpression of *MdWOX13-1* in apple increases the ROS scavenging capacity to cope with drought [55]. In this study, it was also found that abiotic stresses (4 °C, 42 °C, NaCl, PEG) and hormone treatments (MeJA, GA_3_, SA, and NAA) either induced or suppressed *CpWUS* expression to varying degrees (Figure 5c–j).

*WUS* plays a crucial role in flower differentiation and floral organ formation during FM development. By maintaining the stem cell population in the floral meristem, *WUS* ensures normal flower development and organ formation [56]. In the *wus*, the SAM fails to produce new organs and is quickly depleted [3]. During flower development, *WUS* expression is shut down at the sixth stage of flower development, coinciding with the formation of the pistil primordium, followed by the termination of the FM [57]. Studies have found that many genes regulate *WUS* expression and play roles in the determination of FM. As an integrator, the WUS plays an important role in determining the floral meristems. In cucumbers, *CsWUS* expression was detected in the sub-apical region of the SAM and FM, and the overexpression of *CsLFY* led to the upregulation of *WUS*, *AP1*, and *AG* [25], whereas *LFY* was induced in the same tissues where *WUS* was overexpressed [58]. In this study, the overexpression of *CpWUS* promoted the expression of endogenous genes such as *AP1*, *FT*, and *FD* in tobacco, leading to early flowering. The *WUS* also plays an important role in anther development. The negative feedback loop formed by the interaction between WUS and AG regulates the fate of the floral meristem and determines the identity of inner floral organs [3,21,59]. Overexpression of WUS in chrysanthemums reduces the number of anthers in *Arabidopsis* [60]. In the present study, although there was no change in the number of anthers in tobacco, the expression of the *MADS*-box gene *AG* was significantly suppressed.

Root development in plants is a complex process in which the WOX transcription factor family plays an important role. In *Arabidopsis*, root development is influenced not only by *WOX13* and *WOX14* [26] and the co-regulation of *WOX11* and *WOX12* [35], but also by *WOX5*, which regulates the root apical meristem [33]. In apples, the overexpression of *MdWUS* plants did not significantly affect root length, but significantly increased the number of lateral roots [61]. In the present study, *CpWUS* was highly expressed in the roots, suggesting that it may affect root development. In transgenic *N. benthamiana*, *CpWUS* increased primary root length without affecting the number of lateral roots.

## 4. Materials and Methods

### 4.1. Identification of CpWOX Gene Family, Multiple Sequence Alignment, and Phylogenetic Tree Analysis

Using HMMER V3.0 software, the hidden Markov model (HMM) of the WOX domain (Pf00046) was employed to search for all protein sequences. Potential WOX family protein sequences were submitted to the NCBI CDD online database (https://www.ncbi.nlm.nih.gov/Structure/cdd/wrpsb.cgi, accessed on 7 November 2024) for domain screening. ExPasy (http://web.expasy.org/protparam/, accessed on 7 November 2024) was used to determine the primary physical properties of the WOX proteins. Cell-PLoc 2.0 software (http://www.csbio.sjtu.edu.cn/bioinf/Cell-PLoc-2/, accessed on 7 November 2024) was used to predict the subcellular localisation. The genes were named *CpWOX1-12* and *CpWUS* according to their chromosomal locations. MEGA X [62] software was used to compare the WOX sequences. A phylogenetic tree of the WOX family protein sequences from wintersweet, Arabidopsis [8], rice [8], and apple [44] was constructed using the neighbour-joining method.

### 4.2. Gene Structure, Protein Structure, and Promoter Cis-Acting Element Analysis

The CDS sequences and annotation files of the wintersweet WOX family genes were extracted using TBtools Fasta Extract, and gene structure diagrams were visualised using TBtools software [63]. The MEME suite online website (https://meme-suite.org/meme/, accessed on 7 November 2024) was used to analyse conserved motifs, with the number of motifs set to six. MEME analysis results were downloaded and visualised using the TBtools Gene Structure View tool. The TBtools Gf/Gff3 Sequence Extract tool was used to extract promoter sequences 2000 bp upstream of the ATG of the wintersweet *WOX* family genes. Cis-acting element analysis was performed using the PlantCARE website (https://bioinformatics.psb.ugent.be/webtools/plantcare/html/, accessed on 7 November 2024), and the results were filtered to select cis-elements related to stress, hormones, plant defence, growth, and development and visualised using TBtools Simple BioSequence Viewer.

### 4.3. Chromosomal Localisation and Synteny Analysis

First, gene density information was extracted, and a chromosomal location report was generated using the gene location visualisation tool. Based on the genomic information of each species, TBtools software [63] One-Step MCScanX plugin was used to identify syntenic blocks between wintersweet and Arabidopsis, rice, *Chimonanthus salicifolius*, apple, and grape. Gene synteny was visualised using the Dual Synteny Plot plugin (TB tools).

### 4.4. Cloning of CpWOX

Total RNA was extracted from mixed flower samples at different developmental stages of five-year-old wintersweet using an EASYspin Plant RNA Rapid Extraction Kit (Boer, Beijing, China). First-strand cDNA was synthesised using the PrimeScript RT Reagent Kit with gDNA Eraser (TaKaRa, Dalian, China), following the manufacturer’s instructions. The CDS of *CpWUS* was amplified using a PfuDNA Polymerase Kit (TransGen, Beijing, China) and sequence-specific primers (Table S1). PCR products were cloned into the pMD19-T vector (Takara, Shiga, Japan) for sequencing.

### 4.5. Transcriptional Self-Activation Activity and Subcellular Localisation

The self-activation activity of CpWUS was detected using the Matchmaker^®^ Gold Y2H system. The CDS of *CpWUS* was inserted into the pGBKT7 vector and co-transformed with the empty pGADT7 into the yeast strain Y2H Gold. The pGBKT7-Lam and pGADT7-T plasmids were used as negative and the pGBKT7-53 and pGADT7-T plasmids were used as positive controls. The self-activation activity was tested by culturing on SD/−Ade/−His/−Leu/−Trp medium containing X-α-Gal.

To determine the subcellular localisation of CpWUS, an open reading frame (ORF) without a stop codon was cloned into the 1300-GFP vector, which contained a GFP reporter gene controlled by the CaMV 35S promoter. The fusion construct was integrated into *Agrobacterium tumefaciens* strain GV3101. The fusion construct or control vector were used to infect tobacco leaves. Red fluorescent protein (RFP), a nuclear marker (VirD 2NLS-mCherry), was co-transfected to localise to the nucleus. After infiltration, the plants were incubated in the dark for 1 d and then in light for 1 d before the fluorescence signal was detected using a laser scanning confocal microscope.

### 4.6. Gene Expression Analysis

Gene expression analysis was performed using qRT-PCR with SsoFastTM EvaGreen^®^ Supermix and the Bio-Rad CFX 96 system. To analyse the expression pattern of the *CpWUS* gene in *C. praecox*, samples were collected from different stages and tissues of wintersweet, including roots, stems, leaves, and floral organs (middle/inner tepals, stamens, and pistils) of wintersweet grown in the germplasm resource nursery at Southwest University. Additionally, six-leaf stage plants were used as experimental materials to examine *CpWUS* expression under treatments of 4 °C, 42 °C, 300 mM NaCl, 50% PEG-6000, 300 mM SA, 500 mg/L GA_3_, 100 mM MeJA, and 200 mg/L NAA, with untreated samples serving as CK. All collected tissues were immediately frozen in liquid nitrogen. The qRT-PCR primers used are listed in Supplementary Table S1. The qRT-PCR was carried out under the following conditions: 3 min at 95 °C, followed by 40 cycles of 5 s at 95 °C, 5 s at 60 °C, and 5 s at 72 °C, along with a melt curve from 65 °C to 95 °C. Cp18S and CpRPL8 were used as reference genes for the normalisation of wintersweet data [64]. NbActin was used as an internal reference for the normalisation of tobacco data (Table S1). Gene expression levels were analysed using the 2^−ΔΔCT^ method [65].

### 4.7. Generation of Transgenic Tobacco

The genetic transformation of tobacco was performed using the leaf-disk method [42]. Tobacco seeds were soaked in sterile water with 5% sodium hypochlorite (NaClO) at a 5:1 ratio for 10 min, then washed 4–5 times and transferred to MS medium. Tobacco leaves were cut into 0.5 × 0.5 cm pieces and pre-cultured for 2 days on MS + 2.0 mg/L 6-BA + 0.2 mg/L NAA medium. The 35S::*CpWUS* transformed GV 3101 strain was cultured in YEB liquid medium to an OD600 of 0.8, then resuspended in an infection solution (MS + 50 mg/L AS + 50 mg/L MES) to an OD600 of 0.4. After pre-culturing the tobacco leaf disks, they were shaken gently in the dark for 10 min and transferred to MS + 2.0 mg/L 6-BA + 0.2 mg/L NAA + 500 mg/L Cb + 10 mg/L hygromycin medium for selection. PCR was used to check for the presence of the construct in the transgenic lines and qRT-PCR was performed to verify its expression. Transgenic seeds were screened using 25 mg/L hygromycin (hyg). After 10–15 days, the seedlings were transferred to nutrient pots (vermiculite: peat = 1:1) and grown in a controlled growth chamber (25 °C, 16 h light/8 h dark photoperiod, 2000 Lux light intensity, 70% relative humidity).

### 4.8. Statistical Analysis

Data were statistically analysed by one-way analysis of variance (ANOVA) and Duncan’s test using the IBM SPSS 22 software (SPSS, Chicago, IL, USA). The values of *p* < 0.05 and *p* < 0.01 were recognized as statistically significant and extremely significant, respectively.

## 5. Conclusions

The 13 *WOX* genes identified in wintersweet were evolutionarily classified into WUS, ancient, and intermediate branches, all containing the conserved homeobox domain. The promoter regions were enriched with cis-acting elements related to hormone response, stress response, and development, suggesting their functional diversity. *CpWUS* encodes a nuclear-localised transcription factor with self-activation activity, is highly expressed in anthers and roots, and is induced by low temperatures, high temperatures, salt, and MeJA. This suggests that *CpWUS* may integrate environmental signals and hormonal pathways to regulate developmental processes. *CpWUS* overexpression promotes tobacco flowering by upregulating floral genes (*NbFT, NbAP1*, and *NbFD*) and primary root elongation. These findings provide a new perspective for functional studies of *WOX* genes in woody plants. As a candidate gene for regulating flowering time and root system architecture, *CpWUS* can serve as a target for molecular breeding and improvement of stress resistance in wintersweet.

## Figures and Tables

**Table 1:** Physicochemical properties of CpWOX family proteins in wintersweet.

Sequence ID	Gene Name	Amino Acid	Molecular Weight	Theoretical pI	Instability Index	Aliphatic Index	Grand Average of Hydropathicity	Subcellular Location
Cpra01G00013.1	CpWOX1	159	18669.12	8.62	56.15	71.7	−0.798	Nucleus
Cpra02G01741.1	CpWOX2	185	21441.46	9.05	54.1	55.35	−0.839	Nucleus
Cpra03G00351.1	CpWOX3	207	23340.11	6.66	49.71	65.99	−0.821	Nucleus
Cpra03G01103.1	CpWOX4	313	35020.28	8.93	63.2	61.47	−0.583	Nucleus
Cpra04G00358.1	CpWOX5	214	24178.15	9.13	54.81	60.19	−0.943	Nucleus
Cpra05G00919.1	CpWOX6	192	21821.53	5.79	62.76	66.51	−0.827	Nucleus
Cpra06G01843.1	CpWOX7	346	37937.55	6.51	61.19	66.24	−0.539	Nucleus
Cpra08G00857.1	CpWOX8	311	34981.89	8.48	67.5	60.29	−0.727	Nucleus
Cpra08G00936.1	CpWOX9	236	26214.51	5.25	76.21	76.78	−0.424	Nucleus
Cpra09G00234.1	CpWOX10	235	26738.95	6.97	64.77	58.81	−0.762	Nucleus
Cpra09G00715.1	CpWOX11	181	20892.75	8.52	54.47	56.08	−0.851	Nucleus
Cpra11G00217.1	CpWOX12	279	31555.08	5.68	65.45	60.47	−0.92	Nucleus
Cpra11G00833.1	CpWUS	270	30006.96	5.86	54.08	49.11	−0.857	Nucleus

## Data Availability

Data are contained within the article and Supplementary Materials.

## Supplementary Materials

The following supporting information can be downloaded at: https://www.mdpi.com/article/10.3390/plants14071144/s1, Table S1: List of primers.
