# Supplementary material for: Genome-Wide Identification of WOX Gene Family in Chimonanthus praecox and a Functional Analysis of CpWUS
Source: Plants (Basel). 2025 Apr 7;14(7):1144. doi: 10.3390/plants14071144 (PMC11991195; doi:10.3390/plants14071144)
Supplement: Supplementary file 1 [file plants-14-01144-s001.zip › plants-3549486-supplementary.pdf]

Table S1 List of primers

Primer name	Primer sequences (5′-3′)
CpWUS-F	CTCTCCTACATGGAACCTCAGCAAT
CpWUS-R	GAGGCACGTGATATCAACAACCAGG
1300-*CpWUS* -F	cacgggggacgagctcATGGAACCTCAGCAATACCAGC
1300-*CpWUS* -R	ctcaccatgtcgactctagaACAACCAGGCCACGTGGG
BD-*CpWUS* -F	tggccatggaggccgaattcATGGAACCTCAGCAATACCAGC
BD-*CpWUS* -R	cgctgcaggtcgacggatccACAACCAGGCCACGTGGG
qCp18S-F	CTTTGCCTGATGATGGGATT
qCp18S-R	GCAAAAGGTCACCATCCACT
qCpRPL8-F	AAGATTGTACCAAGTGGTTGC
qCpRPL8-R	TGCTCTACAGGGTTCATAGC
qCpWUS -F	GAACCTCAGCAATACCAGCA
qCpWUS -R	TGGTCAGTAGTGGGAATCCA
qNbActin-F	TGCAAAGACCAGCTCTTCTG
qNbActin-R	ATTCCTGCAGCTTCCATTCC
qNbFT-F	GTCAAGAATCGACCCTTTAGTAG
qNbFT-R	TGAACCCTAGGTTGCTTAAC
qNbAG-F	GATGAACTTGATGCCAGGGAGTT
qNbAG-R	GTCTAGTGTAATGGTTGTTGGATTGC
qNbFD-F	CATCATCCACATCTTCATCTC

qNbFD-R	CTAGAAGTAGTAGTGTGGTC
